# Epithelial–Mesenchymal Transition in the Pathogenesis of Idiopathic Pulmonary Fibrosis

**DOI:** 10.3390/medicina55040083

**Published:** 2019-03-28

**Authors:** Francesco Salton, Maria Concetta Volpe, Marco Confalonieri

**Affiliations:** 1 Pulmonology Department, University Hospital of Cattinara, 34149 Trieste, Italy; marco.confalonieri@asuits.sanita.fvg.it; 2 Life Sciences Department, University of Trieste, 34127 Trieste, Italy; maria.volpe@icgeb.org

**Keywords:** idiopathic pulmonary fibrosis, epithelial mesenchymal transition, myofibroblasts, UIP, lung repair

## Abstract

Idiopathic pulmonary fibrosis (IPF) is a serious disease of the lung, which leads to extensive parenchymal scarring and death from respiratory failure. The most accepted hypothesis for IPF pathogenesis relies on the inability of the alveolar epithelium to regenerate after injury. Alveolar epithelial cells become apoptotic and rare, fibroblasts/myofibroblasts accumulate and extracellular matrix (ECM) is deposited in response to the aberrant activation of several pathways that are physiologically implicated in alveologenesis and repair but also favor the creation of excessive fibrosis via different mechanisms, including epithelial–mesenchymal transition (EMT). EMT is a pathophysiological process in which epithelial cells lose part of their characteristics and markers, while gaining mesenchymal ones. A role for EMT in the pathogenesis of IPF has been widely hypothesized and indirectly demonstrated; however, precise definition of its mechanisms and relevance has been hindered by the lack of a reliable animal model and needs further studies. The overall available evidence conceptualizes EMT as an alternative cell and tissue normal regeneration, which could open the way to novel diagnostic and prognostic biomarkers, as well as to more effective treatment options.

## 1. Introduction

Idiopathic pulmonary fibrosis (IPF) is a specific form of interstitial pneumonia that leads to progressive, irreversible scarring of the lung and death due to respiratory failure within five years in approximately 50% of the patients [1]. Survival rate has not been improved by the recent introduction of two antifibrotic drugs, thus lung transplantation remains the only effective treatment [2]. Clinical and functional worsening are generally proportional to the spreading of the histopathological pattern UIP (usual interstitial pneumonia), which is characterized by patchy areas of dense fibrosis with basal and subpleural predominance causing extensive remodeling of lung architecture [3,4]. Hallmarks of UIP are the presence of areas of mesenchymal cells surrounded by extracellular matrix (fibroblast foci), the hyperplasia of alveolar type-II cells (AT-II) and the absence of inflammatory infiltrates [3].

IPF prevalence is increasing worldwide and incidence increases with age, suggesting that senescence-related mechanisms could be major drivers in the pathogenesis of the disease [1,5,6]. Several associated risk factors and genetic defects have been described in both familial and sporadic cases. Particularly, several familial cases of pulmonary fibrosis exhibit mutations in genes normally expressed by AT-II cells (e.g., Surfactant proteins, Mucin-5B, and ATP-binding cassette A3) [1]. The current paradigm considers alveolar epithelial cells as central players in the pathogenesis due to reduction of their regenerative potential [7]. Alveolar epithelial type-II cells (AT-II) are facultative progenitor cells in normal lung and allow regeneration of the alveolar epithelium via trans-differentiation into alveolar type-I cells (AT-I) after injury [8,9]. It has been suggested that epithelial cells in IPF lung are not able to fulfill this stem-like process, leading to apoptosis and favoring excessive deposition of extracellular matrix (ECM), which eventually causes fibrosis [1,4,7,10].

**Figure 1:**
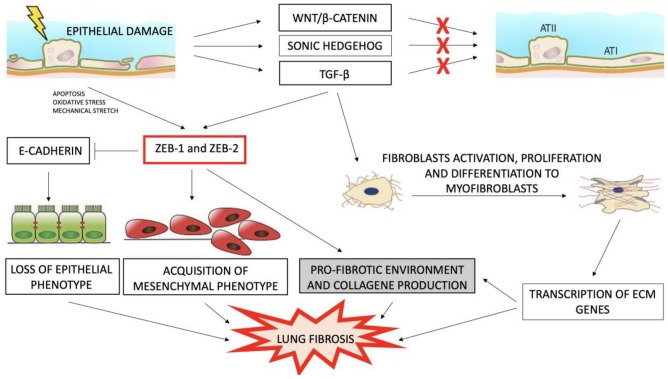
Key pathways regulating lung fibrosis. Repetitive injuries of lung lead to an aberrant activation of developmental/EMT (epithelial–mesenchymal transition) pathways (e.g., Wnt, Sonic Hedgehog and transforming growth factor-beta (TGF-β)) due to the inability of the alveolar epithelium to regenerate. This creates a pro-fibrotic environment in which loss of epithelial phenotype, acquisition of mesenchymal phenotype, fibroblasts activation and collagen production take place. The combination of these events leads to lung fibrosis.

Similar to other organs, normal healing of the lung requires a coordinated response that leads to repair of the barrier integrity through formation of a provisional matrix, myofibroblasts migration and wound contraction, followed by epithelial regeneration of the damaged area, remodeling and removal of debris and extracellular matrix [11,12]. On the contrary, stem cell exhaustion in IPF lung seems to drive abnormal repair and failure of alveolar regeneration with aberrant expression of Wnt/β-catenin and other developmental pathways [13,14] (Figure 1). This creates a profibrotic environment in which collagen-producing fibroblasts and myofibroblasts accumulate through different mechanisms such as proliferation and differentiation of resident lung fibroblasts, transition of bone-marrow derived fibrocytes or other circulating progenitors to fibroblasts and epithelial-to-mesenchymal transition (EMT) [15,16].

## 2. Epithelial–Mesenchymal Transition

EMT is a biological process in which epithelial cells lose contact adhesion and apical-basal polarity, alter their shape with dramatic cytoskeletal changes and acquire some mesenchymal features of invasion, migration and production of ECM [17,18]. EMT is a physiological and often reversible process necessary for normal embryonic development, but it also occurs during response to injury, carcinogenesis and fibrosis [14,18]. However, its precise role in adult pathological states remains elusive [18]. The presence of EMT is defined by the detection of several biomarkers that mirror the loss of epithelial phenotype and the gain of mesenchymal one, namely proteins involved in cell contact (loss of E-cadherin and gain of N-cadherin), cytoskeletal proteins (loss of cytokeratins and gain of vimentin, α-smooth muscle actin, desmin, and fibronectin) and luminal proteins secreted by the original cells (e.g., loss of surfactant production and gain of extracellular matrix or metalloproteinases secretion) [14].

Three different functional categories of EMT are traditionally recognized: type I is associated with physiological processes involved in tissue and organ formation during embryogenesis; type II refers to normal wound healing and plays a role in excessive tissue repair as seen in IPF; type III indicates the acquisition of a migratory phenotype by malignant epithelial cells associated with tumor invasiveness and metastasis [19].

EMT is regulated by multiple extracellular ligands, such as transforming growth factor-beta (TGF-β), epidermal growth factor (EGF), fibroblast growth factor (FGF), interleukin-1 (IL-1), connective tissue growth factor (CTGF), insulin-like growth factor-2 (IGF-2), nuclear factor-kB (NF-kB) and Wnt, that initiate intracellular signaling cascades after binding to surface receptors [18,20]. These pathways can activate one or more EMT-driving transcription factors such as SNAIL1, SNAIL2, TWIST1, ZEB1 and ZEB2, which directly or indirectly downregulate the expression of adhesion molecules such as E-cadherin [20]. Additionally, cell environment and pleiotropic signals such as reactive oxygen species play a role in different signaling pathways leading to EMT [20,21] (Figure 1).

TGF-β is one of the most studied growth factors involved in EMT. After binding, it induces dimerization of two receptors and subsequent auto-phosphorylation, leading to activation of SMAD2 and SMAD3. The SMAD2/3 dimer forms a complex with SMAD4 that translocates into the nucleus and participates in the transcriptional regulation of target genes [22]. The SMAD complex then represses the expression of E-cadherin through SNAIL1 and SNAIL2 transcription factors, which induce the expression of mesenchymal proteins such as N-cadherin, fibronectin and metalloproteinases. Furthermore, TGFβ/SMAD signaling drives the EMT transcription response indirectly through induced expression of TWIST, ZEB1 and ZEB2, and overlaps with other EMT pathways such as Wnt [22,23].

Wnt/Frizzled is a major EMT-driving signal that leads to stabilization of β-catenin and is strongly involved in lung remodeling mechanisms under pathological conditions, [13]. β-catenin activates its own target genes through interactions with TCF/LEF [24] and it has emerged as an important SMAD coactivator. Briefly, the ligand Wnt binds to the receptor Frizzled (Fzd) inducing its phosphorylation. This contributes to the cytosolic accumulation of β-catenin and translocation into the nucleus, where it interacts with specific transcription factors, causing modification of cytokeratin expression and reorganization of the cytoskeleton [14,25]. The canonical Wnt pathway has also been described as a mediator of TGF-β signaling in alveolar type-II cells and pulmonary fibrosis [23,26,27].

Other signaling pathways such as Notch, nuclear factor-kB and Sonic hedgehog (Shh) have been shown to participate in EMT at different levels, promoting transcriptional changes that lead to loss of the adherens junction complex, breakdown of the apical-basal polarity, and cytoskeletal rearrangement [20,28].

## 3. The Role of EMT in Idiopathic Pulmonary Fibrosis

While the definition of EMT traditionally encompassed the direct conversion of epithelial cells into mesenchymal fibroblasts, it has recently evolved to include modest changes in epithelial morphology, motility, and gene expression at a tissue level [20].

In particular, lung fibrosis has long been categorized as a type II EMT event [29,30], but the cellular networks that contribute to tissue scarring have not been well characterized in humans. Type II EMT is physiologically induced in response to injury and stops when tissue repair leads to wound healing and subsequent regeneration [19]. During fibrosis, the persistence of EMT-inducing signals generates ECM accumulation causing tissue remodeling and organ pathology [31]. This was confirmed by in vitro studies showing that TGF-β can induce EMT in human AT-II cells in a time and concentration dependent manner [32,33]. However, in vivo occurrence of EMT in IPF is controversial, as lineage tracing studies report conflicting results, either supporting or denying a pathogenetic role for EMT in lung fibrosis [24,34]. For example, Rock et al. [35] followed AT-II cells fate in a bleomycin-induced murine transgenic model of pulmonary fibrosis and found no transition of labeled AT-II cells into myofibroblasts. Conversely, in the past decade, experimental research publications show that AT-II cells undergo EMT during bleomycin-induced pulmonary fibrosis [34,36] but those studies have been criticized [35] because of poor evidence. It must be highlighted that the demonstration of pathobiological processes in bleomycin-induced murine models of pulmonary fibrosis may not fit human IPF for several reasons: in fact, animal models develop reversible fibrosis preceded by high levels of inflammation, which mimics neither the histological appearance nor the progressive behavior of IPF [37,38]. The typical histological finding in UIP is the presence of heterogeneous areas of progressive scarring, departing from the basal and lateral parts of the lung and leading to extensive remodeling and organ disfunction [39]. A hallmark of these scarred areas are fibroblast foci, collections of fibroblasts and myofibroblasts (α-SMA positive) that actively produce extracellular matrix [3]. While the source of fibroblast foci has been debated for a long time [31], mesenchymal-derived cells in IPF are often found to co-express epithelial and mesenchymal markers, denoting an incomplete transition [40], which has been labeled as partial EMT [41,42].

Some of the pathways involved in EMT are known to play an essential role in normal lung development and regeneration after damage. Recent evidence shows that individual AT-II^Axin2+^ stem cells reside in single-cell fibroblast niches providing juxtacrine Wnt signaling that maintains them as stem cells during homeostasis [43]. Lung injury induces the secretion of autocrine Wnt, allowing proliferation in response to mitogens and expansion of the progenitor pool [43,44]. It has been suggested that the inability of exhausted AT-II to trans-differentiate into AT-I and to regenerate the epithelial layer after continuous damage may cause aberrant activation of the physiological signaling pathways upstream of this process [10,16,45], which eventually leads to fibrosis. Even if direct confirmation is lacking, evidence in support of this hypothesis comes from immunohistochemistry staining of IPF lung specimens showing aberrant activation of major developmental pathways such as canonical Wnt/β-catenin, TGF-β, Zinc Finger E-Box Binding Homeobox1 (ZEB1) and β-tubulin-III (Tubβ3) [13,23]. Noteworthily, all these proteins are partially under the negative control of the microRNA-200 family [23,46]. The Wnt pathway is mainly activated by the alveolar epithelium that is not able to complete the regeneration process. Wnt both activates fibroblasts and interacts with TGF-β, a key mediator of the fibrotic process [47]. TGF-β mediates EMT directly via SMAD-operated suppression of epithelial genes and expression of mesenchymal ones, or indirectly inducing the expression of several transcription factors such as Twist and ZEB [22]. Additionally, TGF-β mediates recruitment, activation and differentiation of fibroblasts in myofibroblasts, extracellular matrix production and apoptosis of epithelial cells [21,22].

Convincing evidence has recently been provided that EMT contributes to the early development of interstitial fibrosis via paracrine signaling directed from the alveolar epithelium to underlying fibroblasts [48]. This epithelial–mesenchymal crosstalk is controlled by ZEB1, which is overexpressed in alveolar type-II cells adjacent to sites of extracellular matrix (ECM) deposition. Persistent activation of ZEB1 may create a profibrogenic microenvironment that leads to the development of fibrosis through an EMT process primarily based on paracrine mechanisms rather than on the direct conversion of epithelial cells into mesenchymal ones [48].

All these pathways converge to EMT, which may be a chief pathogenic mechanism leading to pneumocyte loss, myofibroblast accumulation and lung fibrosis. Aberrant EMT can also be directly triggered by ageing-related mechanisms, including alveolar epithelial cell injury alone, ER stress, unfolded protein response, overexpression of TGF-β and premature apoptosis of ATII cells [15,31]. Particularly, the secretome of apoptotic alveolar cells seems to be a direct driver of EMT through ZEB1 activation [49], which is enhanced by fibroblast-derived TGF-β production. It is also known that mechanical stress is a possible inducer of EMT [50,51]. Since there is a correspondence between the peripheral localization of early lesions in IPF and the anatomical distribution of mechanical stress during respiratory movements [52], it has been argued that a deranged activation of lung reparative processes by mechanical stress may contribute to progressive lung remodeling in IPF via EMT [53].

Similarly, calretinin-positive pleural mesothelial cells (PMC) have been demonstrated in explanted IPF lung tissue and their number correlates with the degree of fibrotic change. This observation suggests that trafficking PMCs could play a pathogenetic role in IPF via mesothelial–mesenchymal transition and invasion of the lung in a centripetal fashion under several stimuli [54,55].

## 4. Conclusions

Even if the detailed pathogenetic mechanisms of IPF are not completely understood, most studies currently acknowledge that EMT occurs to some extent [23,24,40,41,48]. Much of the uncertainties regarding EMT in human pathology probably come from ambiguities in its own definition, which mainly relies on loss of epithelial and gain of mesenchymal markers or activation of different upstream pathways. In fact, what is the lowest expression threshold sufficient to define a process as EMT is discretionary and difficult to set. It is now clear that EMT takes place in developmental organogenesis; furthermore, EMT transcription factors have been shown to be involved in alveolar re-epithelization during normal turnover and in early phases of repair after lung damage. According to the most widely accepted pathogenetic mechanisms of IPF, it seems appropriate to consider the dysregulated EMT process as an opposed response to the proper EMT observed during lung repair and regeneration in healthy subjects. In this context, the aberrant activation of developmental pathways such as Wnt/β-catenin appears as a marker of attempted and failed lung regeneration [47]. Furthermore, alveolar epithelial cell senescence is a direct drive of EMT and mediates, through apoptosis and modification of the extracellular microenvironment, the phenotype of all surrounding tissue.

While further studies are needed to better elucidate the mechanisms and relevance of EMT in IPF in vivo, these observations may serve as a starting point to the future development of diagnostic and prognostic biomarkers or treatment options based on the modulation of the pathogenic factors underlying EMT as a source of pulmonary fibrosis.
